# Validation and cross-cultural adaptation of the *Multiple Sclerosis Intimacy and Sexuality Questionnaire-15* (MSISQ-15) into Spanish

**DOI:** 10.7717/peerj.15138

**Published:** 2023-04-28

**Authors:** Antonio Esteve Ríos, María José Cabañero-Martínez, Silvia Escribano, Frederick Foley, Sofía García-Sanjuán

**Affiliations:** 1 Department of Nursing/Faculty of Health Science, University of Alicante, San Vicente del Raspeig, Alicante, Spain; 2 Ferkauf Graduate School of Psychology, Yeshiva University, Bronx, NY, United States of America

**Keywords:** Multiple sclerosis, Sexuality, MSISQ-15, Neurology

## Abstract

**Background:**

The Multiple Sclerosis Intimacy and Sexuality Questionnaire-15 (MSISQ-15) is a valid and reliable tool to assess the sexuality of people with multiple sclerosis. The objectives of this study were: 1) to cross-culturally adapt and examine the psychometric properties of the MSISQ-15 in the Spanish context and 2) to examine the association between sexual dysfunction and other related factors.

**Methods:**

We conducted a instrumental study. People diagnosed with multiple sclerosis and members of multiple sclerosis associations in Spain were included. The linguistic adaptation of the questionnaire was performed through a translation-back translation procedure. For the psychometric validation, the confirmatory factor analysis was used while the internal consistency was examined by the ordinal alpha test. The construct validity was examined by correlating the results with the Male Sexual Function (FSH), Female Sexual Function-2 (FSM-2), Dyadic Adjustment Scale-13 (EAD-13) and Multiple Sclerosis International Quality of Life Questionnaire (MusiQoL) questionnaires.

**Results:**

A total of 208 participants were included. Both the fit of the Spanish version of the MSISQ-15 to the original scale and the internal consistency were adequate (*α* = 0.89). The construct validity showed correlations with the FSH, FSM-2, and MusiQoL but not with the EAD-13.

**Conclusions:**

The Spanish version of the MSISQ-15 is a valid and reliable tool to assess the sexuality of people with multiple sclerosis in the Spanish context.

## Introduction

Multiple sclerosis (MS) is a chronic neurodegenerative disease that can affect the sexual function of people affected by it (Drulovic, Kisic-Tepavcevic & Pekmezovic, 2020; Delaney & Donovan, 2017). There are studies which established that the presence of problems related to sexuality in patients with MS is greater than for other neurodegenerative diseases and it could be five times more than in the general population (Drulovic, Kisic-Tepavcevic & Pekmezovic, 2020). Specifically, the prevalence of sexual dysfunction has been estimated at 40–80% in women and 50–90% in men with MS (Drulovic, Kisic-Tepavcevic & Pekmezovic, 2020). Among the problems most frequently reported by people with MS are erectile and ejaculation problems in men, lack of lubrication in women, and loss of libido and difficulty reaching orgasm in both sexes  (Celik et al., 2013). Sexual dysfunctions can have notable repercussions on their quality of life, especially because it can influence factors related to mental health (Schairer et al., 2014). Assessing the presence of sexual dysfunction is relevant not only because of its influence on quality of life in these patients (Altmann et al., 2021a) but also to be able to determine the effectiveness of interventions aimed at improving this variable (Lew-Starowicz & Gianotten, 2015). Nevertheless, research has shown that up to 81% of people with MS have not discussed sexuality with their neurologist (Altmann et al., 2021a) or with any health professional in general (Lew-Starowicz & Gianotten, 2015; Tudor et al., 2018). Among the causes of this lack of attention to the sexuality of people with MS is the fact that patients may harbour feelings of shame and discomfort when talking about their sexual experiences and may therefore avoid talking to healthcare professionals for fear of offending them (Tudor et al., 2018). Other barriers that have been identified are the priority placed by professionals on dealing with other symptoms of MS, presence of friends or relatives in consultations, or lack of knowledge on the subject (Tudor et al., 2018).

The lack of training and knowledge among professionals regarding sexuality in people with MS could be resolved by educating health care professionals about the management and evaluation of sexuality in these patients (Tudor et al., 2018). Therefore, for this to happen, questionnaires for measuring sexual function that have been validated in people with MS must be available. In this context, the only reliable and valid tools for assessing sexuality, including all the possible causes of dysfunction in both men and women with MS, are the *Multiple Sclerosis Intimacy and Sexuality Questionnaire-19* (MSISQ-19) and its reduced version, the *Multiple sclerosis Intimacy and Sexuality Questionnaire-15* (MSISQ-15) (Foley et al., 2013). This questionnaire comprises three subscales that address most of the causes of sexual dysfunction in people with MS: primary causes related to involvement of the central nervous system, secondary causes related to the physical aspects of the disease or those derived from its treatment, and tertiary causes derived from sociocultural and psycho-emotional factors (Sanders et al., 2000).

Either of the two aforementioned tools can be used by health professionals, although the 15-item version is shorter and does not show reduced psychometric properties (Foley et al., 2013). Although the MSISQ-15 questionnaire has been validated and adapted to other languages  (Noordhoff et al., 2018; Monti et al., 2020; Przydacz et al., 2021; Tzitzika et al., 2021; Dogan et al., 2022; Lefebvre et al., 2023), it has not yet been adapted and validated for Spanish environments, so there is currently no questionnaire available with which to measure sexuality specifically in people with MS in Spain. Therefore, the objectives of this study were: (1) to cross-culturally adapt and examine the psychometric properties of the MSISQ-15 in the Spanish context and (2) to examine the association between sexual dysfunction and other related factors.

## Materials & Methods

### Design

We conducted an instrumental study to develop an assessment tool, including its cross-cultural adaptation to the Spanish context and analysis of its psychometric properties (Carretero-Dios & Pérez, 2007).

### Variables and questionnaires

The sociodemographic variables included in the data collection questionnaire were age, gender, educational level, marital status, cohabiting status, subtype of MS, mean time since the definitive diagnosis of MS, and sexual orientation (Table 1). In addition, the questionnaires listed below were also used. The authors have permission to use these questionnaires from the copyright holders.

**Table 1:** Sociodemographic variables (*n* = 208).

Variables	N (%)
**Age** (mean ± SD)	44.59 ± 9.788
**Gender**	
Women	135 (64.9)
Men	73 (35.1)
**Education Level**	
Primary (incomplete)	1 (0.5)
Primary (complete)	12 (5.8)
Secondary	29 (13.9)
Vocational training	62 (29.8)
University level	104 (50)
**Marital status**	
Single	59 (28.4)
Married/common-law partner	122 (58.7)
Separated/divorced	25 (12)
Widowed	2 (1)
**Living arrangements of the couple**	
Cohabiting	142 (68.3)
Not cohabiting	20 (9.6)
No partner at present	46 (22.1)
**Sexual orientation**	
Homosexual	12 (5.8)
Heterosexual	185 (88.9)
Bisexual	11 (5.3)
**Number of children**	
0	92 (44.2)
1	48 (23.1)
2	56 (26.9)
3	10 (4.8)
More than 3	2 (1)
**Multiple sclerosis type**	
Relapsing remitting	140 (67.3)
Primary progressive	25 (12)
Secondary progressive	38 (18.3)
Progressive relapsing	5 (2.4)
Time to diagnosis (Mean ± SD)	11.685 ± 8.5052

**Notes.**

(N) absolute frequency. (%) relative frequency. (SD) standard deviation.

#### The Multiple Sclerosis Intimacy and Sexuality Questionnaire-15 (MSISQ-15)

This questionnaire comprises 15 items divided into three domains: primary, secondary, or tertiary causes. Each item is answered on a Likert scale ranging from 1 (never) to 5 (always). The total score varies from 15 to 75 points such that the higher the score, the more influence the disease has on the person’s sexuality (Foley et al., 2013; Monti et al., 2020). All the items are the same for both sexes, except for item 15 which assesses the ability to achieve or maintain a satisfactory erection in men or the lack of vaginal lubrication in women. The questionnaire obtained a high internal consistency score (Cronbach *α*) = 0.923 and showed moderate–low evidence of convergent construct validity with other scales: the *Patient Determined Disease Steps* (*r* = 0.440), mental health subscale from the Short Form-12 questionnaire (*r* = [−0.27]–[−0.37]) and Performance Scales (*r* = [−0.28]–[−0.54]) (Foley et al., 2013).

#### Female Sexual Function-2 Questionnaire (FSM-2)

This questionnaire, which was developed in Spanish, evaluates sexual dysfunction in women, regardless of their age and sexual orientation  (Sánchez-Sánchez, 2021a). The tool comprises 12 items that form 2 domain types: sexual response evaluators (SREs) and sexual activity descriptors (SADs). The corresponding SREs questions asses the sexual dysfunction and are scored from 1 (greatest dysfunction) to 4 (least dysfunction), except for items 2,3,4,5,6, and 10 which include a score of 0 if there had been no sexual activity in the 4 weeks prior. The lower the score, the higher the level of sexual dysfunction. SADs accounted for 3 items (7,8,9) which were scored from 1 to 4, with lower scores corresponding to a higher risk of clinical impact. The last item consists of a question that allows us to explore whether there has been any event with influence on sex life in the last four weeks and to what grade, not included in the analysis. We calculated the internal consistency for our sample with a Cronbach’s alpha of 0.86 for the domain SREs; being the used in the analyses in this article.

#### Male Sexual Function Questionnaire (FSH)

This scale was developed in Spanish and allows the evaluation of sexual dysfunction in men, regardless of their age and sexual orientation  (Sánchez-Sánchez, 2021b). The FSH comprises 12 items divided into two types of domains: sexual response evaluators (SREs) and sexual activity descriptors (SADs). The corresponding SRE questions are scored from 1 (greatest dysfunction) to 4 (least dysfunction), except for items 3,4,5,6, and 10, which included a score of 0 if there had been no sexual activity in the past 4 weeks. The SADs comprised 3 items (7, 8, 9), of which were scored from 1 to 4, with lower scores corresponding to a higher risk of clinical impact. The last item consists of a question that allows us to describe whether there has been any event with influence on sex life in the last four weeks and to what grade, not included in the analysis  (Sánchez-Sánchez, 2021b). We calculated the internal consistency for our sample with a Cronbach’s alpha of 0.88 for the domain SREs.

#### The Spanish version of the Multiple Sclerosis International Quality of Life questionnaire (MusiQol)

This questionnaire, based on its original version in English (Simeoni et al., 2008), comprises 31 items divided into nine dimensions (activities of daily living, psychological well-being, symptoms, relationships with friends, family relationships, relationship with the health system, sexual and sentimental life, and overcoming and rejection). Each item is answered on a Likert scale ranging from 1 (never/not at all) to 6 (not applicable). Subsequently, the scores for each dimension and the global score are summed and standardised on a scale of 0 to 100. The higher the score, the higher the quality of life (Simeoni et al., 2008). The internal consistency was satisfactory for most of the dimensions (Cronbach *α* = 0.7–0.92), except for the family relationship (*α* = 0.67) and relationship with the health system (*α* = 0.53) dimensions. In terms of convergent validity with other sexual assessment scales, the MusiQoL obtained moderately significant correlations with the Short Form-36 (*r* = 0.496–0.76) and weak–moderate correlations with the 14-Symtom Scale (*r* = −0.24–−0.52) (Fernández et al., 2011).

#### The Spanish version of the Dyadic Adjustment Scale-13 (EAD-13)

This scale, validated from its original version in English (Spanier, 1976), assesses the adjustment of couples. The EAD-13 is a reduced version comprising 13 items divided into three subscales: consensus, satisfaction, and cohesion. The items with five response options are scored from 0 to 4 and those with six response options are scored from 0 to 5. The overall score is obtained by summing all the scores of all the items and the cut-off point is 44 points. The higher the score, the better the fit of the couple. The internal consistency (Cronbach *α*) for the total scale was 0.83 and low and statistically significant correlations (*i.e.,* evidence of convergent validity) were obtained with the *Couple Assertion Questionnaire* (*r* = [−0.29]–[−0.35]) (Santos-Iglesias, Vallejo-Medina & Sierra, 2009).

### Process

The Spanish version of the MSISQ-15 was developed in two phases.

#### Phase 1: Cross-cultural adaptation

A translation from English to Spanish was conducted by two outsources independent native Spanish-language translators. After the first translation, a meeting was held to agree upon the creation of the first version. Subsequently, two other outsources independent native English-language translators conducted the back-translation from Spanish to English. Another consensus meeting was then held to obtain the second version of the questionnaire. Both in the translation and the back translation, the translators evaluated the degree of difficulty they had experienced in adapting the items (on a scale of 1 to 10) and identified the type of change the item had required. Type A indicated that no changes were necessary, type B changes were lexical-semantic alterations or were related to sociocultural expressions, and type C changes denoted items that could not be applied to the language being translated.

We subsequently completed a pilot study of the interpretability and comprehension of the items by conducting interviews. Ten people with MS from MS associations in Elche or Alicante (8 women and 2 men) were interviewed to assess their understanding of the questionnaire and any difficulties they had had in comprehending it. Lastly, a final consensus meeting was held in which the definitive questionnaire for validation was obtained.

#### Phase 2: Analysis of the psychometric properties

The validation sample comprised 208 participants (Table 1), of which 64.9% (*n* = 135) were women and the mean age was 44.6 ± 9.8 years (range = 22–68); 67.3% (*n* = 140) had relapsing-remitting type MS. The mean diagnosis time was 11.7 ± 8.5 years (range = 0–40 years, where 0 represented a same-year diagnosis). Regarding patient sexual orientation, 88.9% (*n* = 185) were heterosexual, 5.8% (*n* = 12 were gay, and 5.3% (*n* = 11) were bisexual. Only the 11% of the participants had one or more comorbid factors such as endocrine disease (3,4%), cardiovascular disease (1,9%), mental health disorders (1,9%) or other illness (5,4%).

The sample was accessed by contacting 82 MS associations located in Spain by email, on up to three occasions, to inform them of the study characteristics and ask for their collaboration. Of these, 13 agreed to participate in this work after being informed of the study objectives, characteristics of the questionnaires, and procedure for completing them. People were included if they had sexual activity over the last 6 months considering the instructions of the MSISQ-15 and if they were cognitively able to respond the form by themselves.

To collect the data, a standardised email was created with the aim that each of the associations would disseminate it to all if their members by email and through their social networks. The emails were sent following a standardised procedure involving three mailings (the first mailing, another one two weeks later, and a third mail three weeks after the initial mail containing the last call to participate). The first email and all the subsequent reminders contained a link to the questionnaire we had prepared on the Google Forms platform as well as a letter presenting the study that was also used to obtain the informed consent of the participants. Prior to collection of the definitive data, the data collection procedure was piloted with two of the associations included in this work with the aim of examining the viability of the procedure and to estimate the response rate obtained.

### Data analysis

SPSS software (version 25.0; IBM Corp., Armonk, NY) was used for all the statistical analyses. The mean and standard deviation were calculated for the quantitative sociodemographic variables and the absolute and relative frequencies were calculated for the qualitative variables. Means and standard deviations were also calculated for each subscale of the MSISQ-15 as well as for the EAD-13, FSH, FSM-2, and MusiQoL questionnaires.

Confirmatory factor analysis was conducted using R software using the weighted least squares means and variance (WLSMV) adjusted estimation method which is appropriate for categorical variables (Rhemtulla, Brosseau-Liard & Savalei, 2012). The following indicators were used to analyse the fit of the data to the model: chi-squared tests; the Tucker–Lewis index (TLI), and comparative fit index (CFI), considering values over 0.9 as acceptable  (Hu & Bentler, 1999) while the value of the root mean square error of approximation (RMSEA) had to be less than or equal to 0.05 (Browne & Cudeck, 1993). The internal consistency was calculated using the ordinal alpha. A value greater than or equal to 0.7 was considered satisfactory (Terwee et al., 2007). We expected the scale to maintain the factorial structure of the original three-dimensional scale, with satisfactory internal consistency values for all the dimensions as well as the total scale.

The construct validity of the tool was examined by calculating the correlations between the dimensions of the MSISQ-15 questionnaire and its global score and the FSH, FSM-2, MusiQoL, and EAD-13 tools, using Spearman’s coefficient, considering values close to ±1 after first verifying that the assumption of data normality had been violated. Values above ±0.7 were considered strong correlations; values between ±0.4 and ±0.6 were moderate, and below ±0.4 were weak (Akoglu, 2018). Our hypotheses were that the MSISQ-15 scores would (1) correlate moderately with the FSH and FSM-2, such that we expected to obtain stronger correlations with the primary causes dimension and weaker correlations with the dimension relative to the secondary causes; (2) MSISQ-15 scores would be significantly associated with EAD-13, albeit at moderately-low levels; and (3) MSISQ-15 scores would show statistically significant although moderate–weak associations with health-related quality of life (MusiQoL), with slightly stronger correlations being obtained with the tertiary causes dimension and weaker correlations being found with the primary causes dimension.

### Ethical considerations

This study was approved by the ethics committee at the University of Alicante (reference number: UA-2021-07-20) and was conducted in accordance with the criteria established in the Declaration of Helsinki. Participation in the study was voluntary, anonymous, and the patients were informed about the possibility of withdrawing from it at any time.

## Results

### Linguistic adaptation

The first translator required type B changes to all the items during the direct translation. The second translator also required type B changes, except for items 1, 3, 12, 13, and 15 which required type A changes. The difficulty of the translation was considered extremely low and so was assigned scores of 1 or 2 for most of the items, apart from item 14 which the second translator assigned a difficulty score of 4. Once the first consensus version was prepared, it was back-translated. Regarding the back translation, type B changes were made to all the items except for 1 and 3, which required type A changes. In terms of difficulty, all the items were scored from 1 to 5 except for item 10, which was given a score of 6. Thus, a second version of the questionnaire in Spanish was agreed upon based on both the back-translations. Finally, in the interviews, the participants did not show difficulties in understanding the questionnaire and rated the difficulty of the items as very low. Therefore, no changes were made to the adapted version of the questionnaire after the interviews.

### Psychometric properties

The results of the CFA with the WLSMV robust estimation method reflected that the structure of the original three-dimension scale of the MSISQ-15 (primary, secondary, and tertiary cause) adequately fit the data (chi squared = 168.11, *p* < 0.001, *df* = 87, TLI = 0.96, CFI = 0.97, and RMSEA = .07 (95% CI [0.052–0.082])). The internal consistency calculated with the ordinal alpha for the overall scale was 0.89; for dimension 1 it was 0.85, for dimension 2 it was 0.81, and for dimension 3 it was 0.87.

The mean scores of all the questionnaires, as well as of each of the MSISQ-15 questionnaire subscales and the MusiQoL are shown in Table 2. Regarding the construct validity, there was evidence of convergent validity with moderate correlations between the MSISQ-15 an FSH (–.52) and FSM-2 (–.55) and, as expected, the strongest correlations were with the primary causes dimension and the weakest ones with the secondary causes dimension. Quality of life was significantly associated with all the dimensions of the MusiQoL and showed moderate-weak correlations with the overall MSISQ-15 score. The strongest correlations were those obtained with the total score (−.39) and with the tertiary causes dimension (−.38). Finally, contrary to our expectations, the MSIQ-15 scores were not significantly correlated with the EAD-13 (Table 3).

**Table 2:** Mean and standard deviation of the MSISQ-15, FSH, FSM-2, and MusiQoL scales.

Scale	Mean (SD)
**MSISQ-15**	32.21 (10.15)
Primary cause	12.31 (4.65)
Secondary cause	9.01 (3.55)
Tertiary cause	10.99 (4.54)
**FSH** (*n* = 73)	17.75 (7.20)
**FSM-2** (*n* =135)	20.82 (5.76)
**EAD-13**	58.95 (9.31)
**MusiQoL**	63.13 (15.41)
ADL	53.13 (25.68)
PWB	49.67 (27.01)
Friends	65.43 (28.94)
Symptoms	54.06 (26.03)
Family	75.48 (31.01)
Health system	77.64 (29.83)
SSQoL	60.76 (32.10)
Coping	56.67 (31.37)
Reject	75.36 (28.63)

**Notes.**

ADL activities of daily living EAD-13 Dyadic Adjustment Scale-13 FSH dimension SERs of Male Sexual Function Questionnaire FSM-2 dimension SERs of Female Sexual Function-2 questionnaire MSISQ-15 Multiple Sclerosis Intimacy and Sexuality Questionnaire-15 MusiQol Multiple Sclerosis International Quality of Life Questionnaire PWB psychological wellbeing SSQoL sentimental and sexual quality of life

**Table 3:** Correlation between the MSIQ-15 scale and its dimensions and the FSH, FSM-2, EAD-13, and MusiQoL questionnaires (Spearman’s R).

	FSH (*n* = 73)	FSM-2 (*n* = 135)	EAD-13 (*n* = 208)	MusiQoL (*n* = 208)
MSIQ-15	−.52^**^	−.55^**^	−.14	−0.39^*^
Primary cause	−.53^**^	−.65^**^	−.14	−.25^*^
Secondary cause	−.31^**^	−.27^**^	−.10	−.35^*^
Tertiary cause	−.42^**^	−.32^**^	−.08	−.38^*^

**Notes.**

FSH dimension SERs of Male Sexual Function Questionnaire FSM-2 dimension SERs of Female Sexual Function Questionnaire-2 EAD-13 Dyadic Adjustment Scale-13 MSISQ-15 Multiple Sclerosis Intimacy and Sexuality Questionnaire-15 MusiQol Multiple Sclerosis International Quality of Life questionnaire

* *p* < 0.01

** *p* < 0.001

## Discussion

In this study we obtained a specific tool in Spanish to measure sexual dysfunction in people with MS. The Spanish version of the MSISQ-15 is a valid and reliable questionnaire that can be used by health professionals to examine sexuality in patients with MS. Although up to 5 scales, including the *Female Sexual Function Index*, *International Index of Erectile Function*, *Sexual Expectations Evaluation in Women with Multiple Sclerosis*, or the *Szasz Sexual Functioning Scale* have been described that could be used to assess sexual dysfunction in people with MS. However, the latter questionnaires only evaluate sexual dysfunctions in one sex or do not address sexuality from the broad spectrum of causes that can lead to problems in people with MS (Carrillo et al., 2020). Therefore, the MSISQ-15 questionnaire and its original 19-item version, the MSISQ-19 (Sanders et al., 2000) that has also been validated in other settings (Nehrych et al., 2019; Silva et al., 2015; Mohammadi et al., 2014; Devis et al., 2022; Carotenuto et al., 2021), could be the best option for assessing sexuality in people with MS because they consider the three possible causes of sexual dysfunction. In turn, this could help guide health professionals in the design and application of interventions aimed at improving the sexuality of patients with MS  (Carrillo et al., 2020).

The CFA showed that the structure of the Spanish version of the MSISQ-15 presented a good fit to the data such that the translation maintained the sense and directionality of the items of the original version and therefore, also showed agreement with the dimensions they intended to evaluate (Foley et al., 2013), as in the French version (Lefebvre et al., 2023). Like the other versions of this questionnaire (Foley et al., 2013; Noordhoff et al., 2018; Monti et al., 2020; Przydacz et al., 2021; Tzitzika et al., 2021; Dogan et al., 2022; Lefebvre et al., 2023), this tool presented an adequate internal consistency with an alpha of 0.89, which was higher than that recommended in the literature (*α* > 0.7) (Browne & Cudeck, 1993).

The convergent analysis confirmed the expected relationship between the MSISQ-15 questionnaire and dimension of SERs of FSH and FSM-2 tools, dimension which assess the sexual dysfunction. This correlation between MSISQ-15 and questionnaires which evaluate sexual dysfunction such as the *Female Sexual Function Index* or the *International Index of Erectile Function* has been obtained in other validation studies of the questionnaire (Noordhoff et al., 2018; Przydacz et al., 2021; Tzitzika et al., 2021; Dogan et al., 2022).

According to our results, the presence of sexual dysfunction seems to be inversely associated with quality of life, so it is possible that the greater the sexual dysfunction, the worse the perceived quality of life. This association between sexual dysfunction and quality of life has also been obtained in other validation studies of the MSISQ-15 which used quality of life questionnaires to evaluate the construct validity (Noordhoff et al., 2018; Dogan et al., 2022). Moreover, this negative relationship has also previously been described in other studies such as those by Tepavcevic et al. (2008), Altmann et al. (2021b), or Vitkova et al. (2014) through quality of life questionnaires which correlated with sexual dysfunction. This influence on quality of life seems to be related to the physical difficulties that people with MS could find when having sexual activity generating in them an impact in mental health related to aspects such as insecurity, anxiety or low self-steem (Schairer et al., 2014; Vitkova et al., 2014). This correlation with psychological aspects was also obtained in the Italian version of the questionnaire (Monti et al., 2020).

Regarding the correlation with the dyadic adjustment of the couple, it seems that there is no relation with the MSISQ-15 questionnaire. Although we found no previous studies that had related these two variables, some work with people with MS found no significant differences in their sexual function according to whether or not they had a partner (McCabe et al., 1996). In addition, despite the presence of sexual dysfunction in people with MS, they and their partners’ levels of satisfaction were not significantly different and so the presence of an alteration in one of the partners should not necessarily influence the sexual interaction (McCabe & McDonald, 2007). This could be related to positive support from partners in the face of the disease, which has been linked to higher sexual satisfaction in people with MS (Blackmore et al., 2011). This lack of correlation could also be explained by changing priorities after a diagnosis of MS whereby sexuality is relegated to the background and attention to other aspects of the disease is prioritised (Koch, Kralik & Eastwood, 2002; Esteve-Ríos et al., 2021).

Among the limitations of this study were the lack of a gold standard questionnaire in Spanish with which to measure sexuality in people with MS. Another limitation was related to the time periods considered by each of the questionnaires, given that the FSH and FSM-2 questionnaires assessed sexuality in the 4 months prior while the MSISQ-15 considered the past 6 months. On the other hand, although we consider the presence of other comorbid factors that could have a relationship on sexuality, they were not included in the analysis. Moreover, although we consider as inclusion criteria having sexual activity over the last six months and being cognitively able to complete the form, it is not possible to assure that all participants met the inclusion criteria. This limitation is intrinsic to the use of online surveys (Andrade, 2020). Finally, in our study, only the MSISQ-15 scale was validated for use in people with MS. However, future work should consider its validation in people with other neurodegenerative pathologies in which its application could also be useful, as shown for other validation studies (Noordhoff et al., 2018; Monti et al., 2020; Przydacz et al., 2021).

## Conclusions

The Spanish version of the MSISQ-15 questionnaire is a valid and reliable questionnaire for assessing the sexuality of people with MS. Thus, health professionals can use this tool not only to assess the presence of sexual dysfunction but also to evaluate the results of any interventions they may implement to try to improve the sexuality of people with MS.

##  Supplemental Information

10.7717/peerj.15138/supp-1 Supplemental Information 1 Dataset: All analysis of sociodemographic characteristics and data obtained from the instruments included in the study

## Additional Information and Declarations

### Data Availability

The following information was supplied regarding data availability: The data are available in the Supplemental Files.
